# Mutant plasminogen in hereditary angioedema is bypassing FXII/kallikrein to generate bradykinin

**DOI:** 10.3389/fphys.2022.1090732

**Published:** 2023-01-05

**Authors:** Stefan Hintze, Britta S. Möhl, Jessica Beyerl, Karin Wulff, Andreas Wieser, Konrad Bork, Peter Meinke

**Affiliations:** ^1^ Friedrich-Baur-Institute at the Department of Neurology, University Hospital, Ludwig-Maximilians-University Munich, Munich, Germany; ^2^ Fraunhofer Institute for Translational Medicine and Pharmacology ITMP, Immunology, Infection and Pandemic Research, Munich, Germany; ^3^ Institute of Virology, School of Medicine, Technical University of Munich/Helmholtz Zentrum München, Munich, Germany; ^4^ Division of Infectious Diseases and Tropical Medicine, University Hospital, Ludwig-Maximilians-University Munich, Munich, Germany; ^5^ Max-von-Pettenkofer Institute, Ludwig-Maximilians-University Munich, Munich, Germany; ^6^ University Medicine, University of Greifswald, Greifswald, Germany; ^7^ DZIF: German Centre for infection research (DZIF), Partner Site Munich, Munich, Germany; ^8^ Department of Dermatology, University Medical Center, Johannes Gutenberg University, Mainz, Germany

**Keywords:** Hereditary angioedema (HAE), normal C1-INH, HAE-PLG, plasminogen, FXII, bradykinin, kallikrein-kinin system (KKS)

## Abstract

Hereditary angioedema (HAE) is characterized by recurrent localized edema in various organs, which can be potentially fatal. There are different types of hereditary angioedema, which include genetic deficiency of C1 inhibitor (C1-INH) and hereditary angioedema with normal C1-INH (HAEnCI). In HAEnCI patients mutations have been identified in the *F12*, *PLG*, *KNG1*, *ANGPT1*, *MYOF*, and *HS3ST6* genes. The release of bradykinin from kininogen *via* the kallikrein-kinin system (KKS) has been shown to be the main mediator in HAE-FXII, but for HAE-PLG there are only first indications how the *PLG* mutations can result in bradykinin release. Here we identified in a multi-generation HAE-PLG family an additional *F12* mutation, resulting in the loss of one *F12* allele. There were no differences in the clinical presentation between HAE-PLG patients with and without the additional *F12* mutation, thus we concluded that the kallikrein-kinin system is bypassed in HAE-PLG. Structural modeling and *in vitro* assays using purified proteins confirmed the *PLG* mutation c.988A>G; p.K330E to be a gain of function mutation resulting in an increased bradykinin release by direct cleavage of high molecular weight kininogen (HMWK). Thus, we can provide clinical and experimental evidence that mutant plasminogen in HAE-PLG is bypassing FXII/kallikrein to generate bradykinin.

## Introduction

Hereditary angioedema (HAE) is an inherited disorder clinically characterized by recurrent and self-limited episodes of localized edema in various organs. Clinical symptoms include skin swellings, abdominal pain attacks, tongue swellings and potentially life-threatening laryngeal attacks. Types of HAE include the classical HAE due to the deficiency of functional C1 inhibitor (HAE-C1-INH), and various new types of HAE with normal activity of C1-INH (HAE with normal C1-INH, HAEnCI). Currently, 6 types of HAEnCI are recognized, based on underlying mutations of factor XII (HAE-FXII) (Dewald and Bork, 2006; Bork et al., 2011), angiopoietin-1 (HAE-ANGPT1) (Bafunno et al., 2018), plasminogen (HAE-PLG) (Bork et al., 2018), kininogen 1 (HAE-KNG1) (Bork et al., 2019), myoferlin (HAE-MYOF) (Ariano et al., 2020), and heparan sulfate-glucosamine 3-O-sulfotransferase 6 (HAE-HS3ST6) (Bork et al., 2021).

Bradykinin, a 9-amino acid peptide chain generated by proteolytic cleavage of its precursor high molecular weight kininogen (HMWK), is considered to be the main mediator of HAE (Kaplan and Joseph, 2010). It is well described how the kallikrein-kinin system (KKS) leads to bradykinin release: binding of negatively charged macromolecules to factor XII induces a conformational change causing activation. As activated factor XII (factor XIIa), it converts prekallikrein (PK) to kallikrein which cleaves HMWK to release bradykinin (Kaplan and Joseph, 2014). The presence of C1 inhibitor prevents this reaction and its deficiency in HAE-C1-INH results in an overproduction of bradykinin (De Maat et al., 2018). Mutations in HAE-FXII can introduce new sites in the mutant FXII protein that are sensitive to enzymatic cleavage by plasmin (de Maat et al., 2016). These FXII mutants rapidly activate after cleavage by plasmin, escape from inhibition through C1-INH, and elicit excessive bradykinin formation. Another study showed that the FXII mutants Thr328Lys and Thr328Arg are cleaved after residue 328 by coagulation proteases like thrombin and FXIa, producing a truncated form of FXII (delta-FXII) which converts PK to kallikrein more efficiently (Ivanov et al., 2019).

In HAE-PLG (Bork et al., 2018), there was a clinical response to a bradykinin B2 receptor 2 antagonist, an indirect sign for bradykinin involvement (Bork et al., 2020). It was unknown whether the KKS cascade pathway or another mechanism is linking the mutant protein to bradykinin overproduction. A recent study did show that plasminogen can directly release bradykinin from kininogens, and more efficiently if carrying the HAE mutation (Dickeson et al., 2022). Our findings in a large family and the following experiments, both presented here, are now shedding some more light on the pathomechanism and prove clinically that the KKS is bypassed for bradykinin production in HAE-PLG.

## Material and methods

### Probands

Probands of this study comprised eight individuals of one four-generation family. All probands came from the Angioedema Outpatient Service (AOS) Mainz. Clinical data was collected from the patients’ medical history and from clinical examinations by one of the authors as well as follow-up records.

### Patient materials and ethics

DNA was extracted from EDTA blood according to standard protocols (Qiagen, Hilden, Germany). For biochemical analysis plasma was separated by centrifugation of freshly collected citrated blood at 1,500 g for 10 min at 4°C. Plasma samples were collected during symptom-free intervals. All subjects analyzed in this study gave written informed consent prior to participation. The local ethic committee approved the study.

### Sanger sequencing

Sanger sequencing was used for the validation of the *PLG* and *F12* mutations. The method was applied as described elsewhere (Bork et al., 2018).

### Biochemical analysis of C1-INH and coagulation systems

All biochemical parameters were tested using citrated patient’s plasma. C1-INH activity in plasma was determined using the chromogenic substrate C_2_H_5_CO-Lys (ε-Cbo)-Gly-Arg-pNA (Technochrom C1-Inhibitor, Technoclone, Vienna, Austria). Plasma levels of C1-INH antigen were assayed by radial immunodiffusion (NOR Partigen C1-INH and C4, Siemens Healthcare Diagnostics, Marburg, Germany). The method used to determine clotting activity of FXII is described elsewhere (Bork et al., 2019).

### Protein assay

For the protein assay mammalian expression vectors for the wild type (wt) and the mutant plasminogen (p.K330E) were produced by VectorBuilder. To obtain relative high amounts of both proteins they were expressed in HEK293 cells. The proteins were purified by size-exclusion chromatography. Both, expression and purification, was done by BioServUK. For the assay two fractions from each protein purification were used. As substrate we used commercially available native single chain HMWK, which was isolated from human plasma (MERCK). HMWK alone or with one of the plasminogens were incubated in 1x PBS, with and without protease inhibitors (Complete ^®^ Ultra Roche # 5892970001) at 37°C. After 22 h an aliquot of each reaction mix was used for the MALDI-TOF analysis. The rest was used for western blot analysis.

### Gel-electrophoresis and western blot

SDS gel electrophoresis with 4%–15% TGX gels (BioRad #456–8087) was used for protein separation. Western Blotting was performed using the TransBlot® Turbo system (BioRad). Proteins were transferred to nitrocellulose membranes (Trans—Blot^®^ Turbo RTA Transfer Kit #170-4270). The membranes were blocked in 5% BSA 1xTBS/0.1% Tween^®^20 buffer. To detect the HMWK or protein fragments we used an antibody directed against the bradykinin sequence (rabbit anti bradykinin, BioRad #0100-0443), as secondary antibody we used Li-Cor’s donkey anti-rabbit IRDye 800CW. Pictures were obtained using the LiCor FC.

### Matrix-Assisted Laser-Desorption/Ionization Time-Of-Flight Mass-Spectrometry (MALDI-TOF-MS)

For MALDI-TOF-MS analysis aliquots of 20 µl of the protein assay solutions were directly desalted and concentrated after the incubation time using C18 ZipTips® (MERCK) according to the manufacturer’s protocol. The samples were eluted from the C18 column material by aspirating and dispensing 5 µl of acetonitrile, .1% trifluoroacetic acid (v/v). 1 µl of the eluates were directly spotted onto a polished steel MALDI-TOF-MS target plate. Dried spots were overlaid with α-HCCA matrix (10 mg/ml α-cyano-4-hydroxy-cinnamic acid, 50% acetonitrile, 2.5% trifluoroacetic acid, v/v, Bruker Daltonik, Bremen, Germany). After drying of the matrix, MALDI-TOF-MS measurements were performed with an autoflex speed LRF mass spectrometer (Bruker Daltonik GmbH, Bremen, Germany) equipped with a 2 kHz Nd:YAG smartbeam-II laser (355 nm). Parameter settings had been optimized for the mass range between 200 and 1500 Da. Spectra were recorded in the positive reflectron mode with the maximum laser frequency. Control measurements were performed with purified bradykinin 1–9 (Echelon Bioscience, Salt Lake City, Utah). A peptide standard mix (Bruker Daltonik GmbH, Bremen, Germany) covering the low mass range was used for instrument calibration. Data visualization and baseline subtraction of the acquired spectra was done by flexAnalysis 3.4 (Bruker Daltonik GmbH, Bremen, Germany).

### Structural analysis of the mutant protein

The structural views were performed using the PyMOL Molecular Graphics System, Version 2.4 Schrödinger LLC.

## Results

**FIGURE 1:**
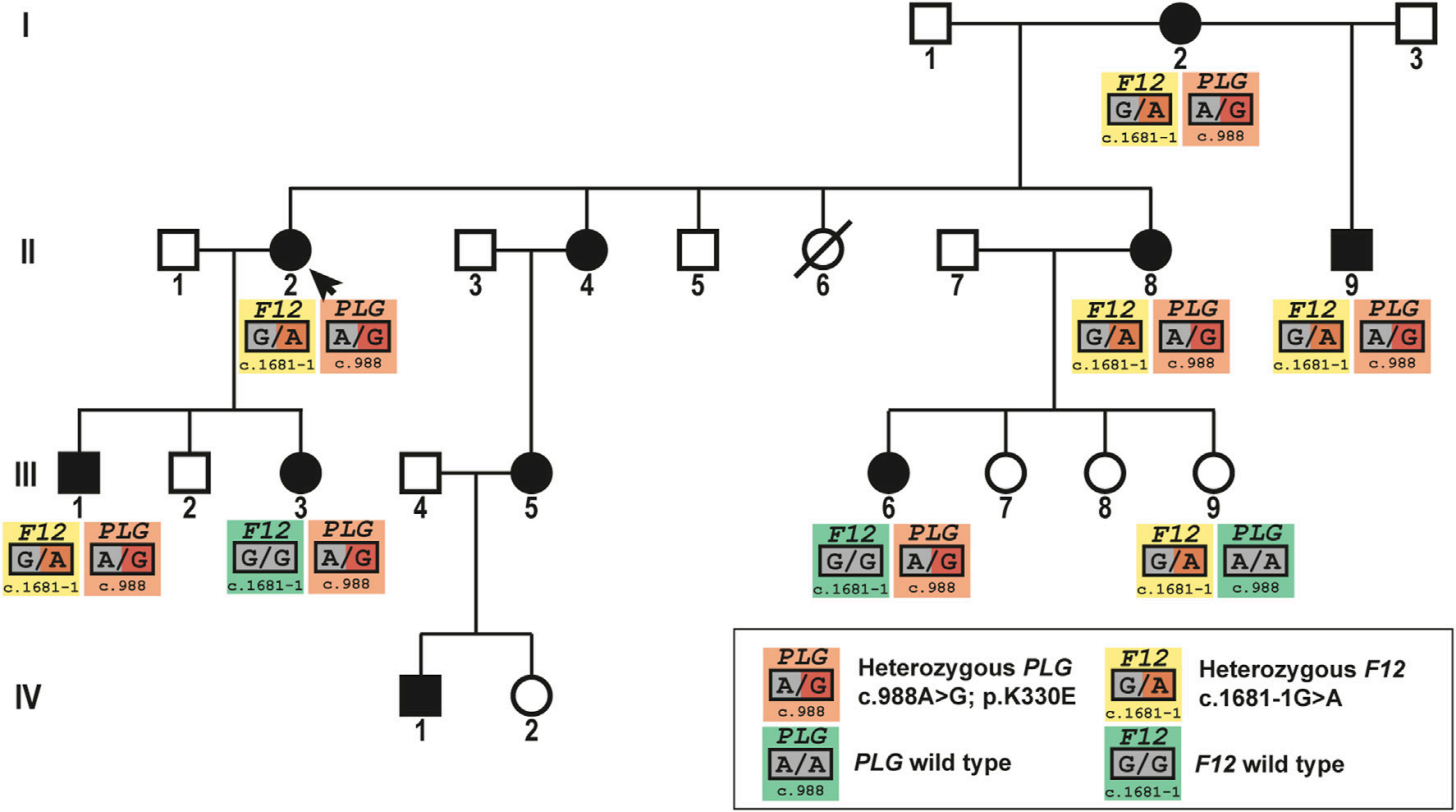
Pedigree of the large HAE-PLG family showing the inheritance of the HAE-causing *PLG* mutation c.988A>G; p.K330E and the *F12* mutation c.1681-1G>A resulting in the loss of protein expression from the mutated allele.

Here, we investigated a four-generation HAE-PLG family, in which affected family members carry the *PLG* mutation c.988A>G; p.K330E. Ten out of 23 individuals in this family were affected by HAE, DNA of seven of these affected individuals was sequenced. In these seven HAE patients the *PLG* mutation c.988A>G; p.K330E was identified. In addition to the HAE-causing *PLG* mutation, we identified an additional pathogenic mutation in the *F12* gene in 6 family members (Figure 1). This additional *F12* mutation (c.1681-1G>A) is not resulting in HAE, but has been identified in FXII deficiency (Schloesser et al., 1995). The *F12* mutation was identified in five HAE patients (in combination with the *PLG* mutation) and one unaffected family member (wild type *PLG*) (Figure 1).

**FIGURE 2:**
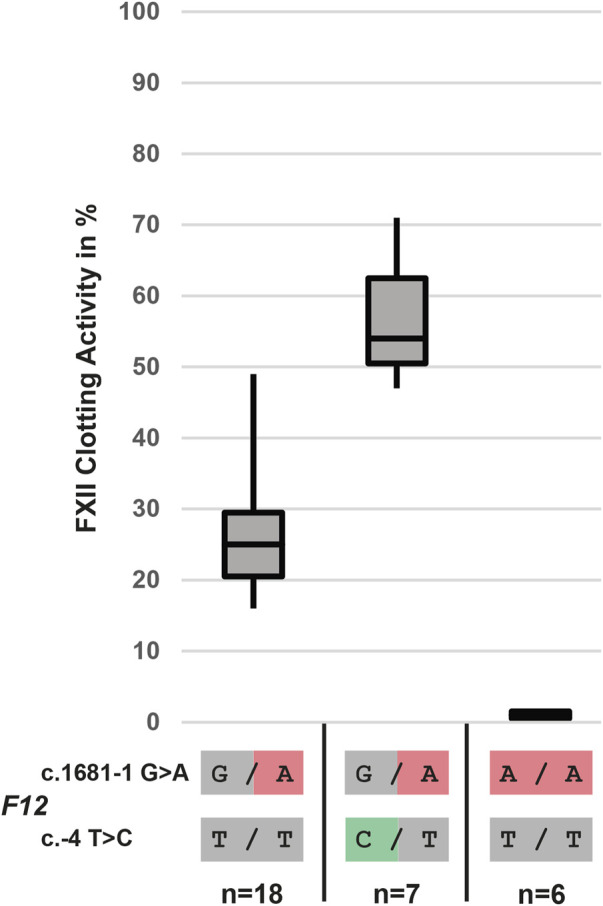
Clotting activity of the *F12* mutation c.1681-1G>A in combination with the c.-4C/T polymorphism in 31 unrelated individuals illustrating the loss of FXII protein.

Individuals carrying the *F12* mutation c.1681-1G>A in a heterozygous state do have, depending on the presence of the -4C/T polymorphism affecting the Kozak region of the *F12* gene on the second allele (Bach et al., 2008), either about 25 or about 50% clotting activity. Individuals homozygous for the *F12* c.1681-1A mutation do have no residual clotting activity (Figure 2).

Considering the role of FXII in the pathomechanism of HAE-C1-INH and HAE-FXII, we concluded that there would be an impact of the loss of one *F12* allele if FXII is involved in the pathomechanism of HAE-PLG. Thus, we performed a clinical investigation based on the presence of the *PLG* and *F12* mutations. We could not identify any differences in the clinical presentation of the five family members carrying the *PLG* mutation c.988A>G; p.K330E and the *F12* mutation c.1681-1G>A compared to the two family members carrying only the *PLG* mutation c.988A>G; p.K330E (Table 1).

**TABLE 1:** Clinical data of affected individuals of the large family with HAE-PLG comparing individuals with and without additional *F12* mutation c.1681-1G>A. (n.a. not available; n.d. not determined).

	Patients with HAE-PLG and *F12* c.1681-1G>A					Patients with HAE-PLG and wild type *F12*	
Patient	I:2	II:2	II:8	II:9	III:1	III:3	II:6
Sex	♀	♀	♀	♂	♂	♀	♀
Age (years)	88	68	63	59	49	35	43
Age of onset	5	20	56	38	n.a	10	25
C1-INH concentration (RR: 16–32 mg/dL)	27.5	26.3	n.d	20.6	17.9	22.5	24.0
C1-INH activity (RR: 70%–130%)	84	88	n.d	73	>125	97	84
Lifetime attacks (estimated total)	835	111	6	>251	0	7	52
Mean attacks per year	10.1	2.3	0.9	12.0	n.a	0.3	2.9
Lifetime attacks per organ (estimated)							
Face	250	1	0	1	0	0	0
Extremities	30	10	0	0	0	0	0
Abdomen	275	0	0	0	0	1	50
Larynx	5	0	0	0	0	0	0
Tongue	275	100	6	>250	0	6	2

**FIGURE 3:**
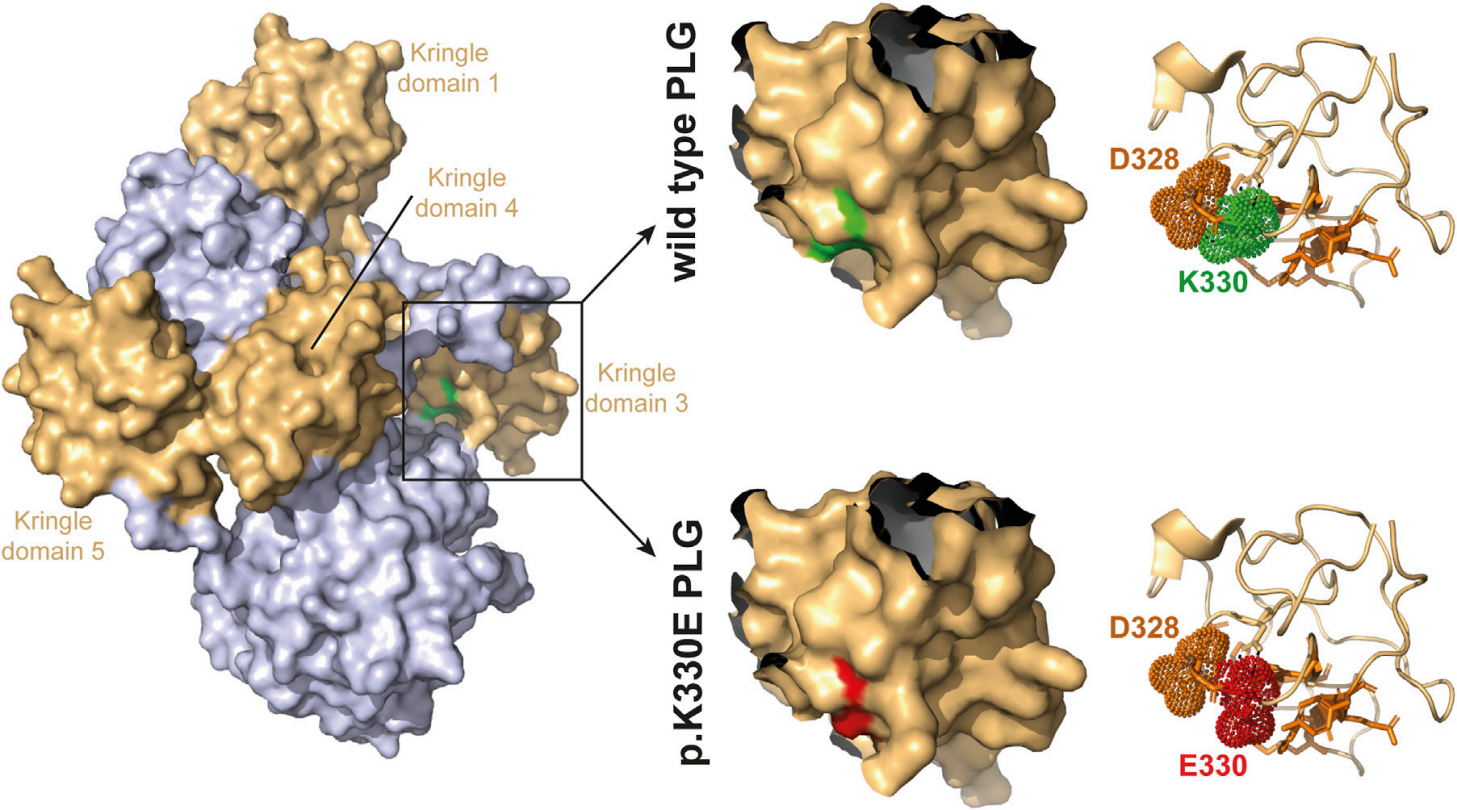
Crystal structure of human plasminogen. The structure is shown in surface (left and middle) and cartoon (right) representations colored in grey with the Kringle domains 1-5 highlighted in light-orange. Cartoon presentations (right) indicate the ligand binding residues as orange sticks, and K330 (green) and the interacting amino acid D328 (orange) as well as the mutation E330 (red) as dots. Structural views of plasminogen (Protein Data Bank entry 4A5T) (Xue et al., 2012) were generated by using the PyMOL Molecular Graphics System, Version 2.4 (Schrödinger LLC).

Based on these results, we concluded that it is possible that mutant plasminogen is not acting *via* the KKS to produce increased amounts of bradykinin. To investigate how the *PLG* mutation is causing HAE we first looked into plasminogen structure. The p.K330E mutation was modeled into the crystal structure of the native plasminogen (Xue et al., 2012). It disrupts the bonding with D328 that is one of the ligand binding residues in Kringle domain 3. The modified side chain and the disrupted D328 bonding cause a widening within the ligand binding groove formed by the hydrophobic surface of this domain (Figure 3). This likely allows for an altered proteolytic activity of the mutant plasminogen.

**FIGURE 4:**
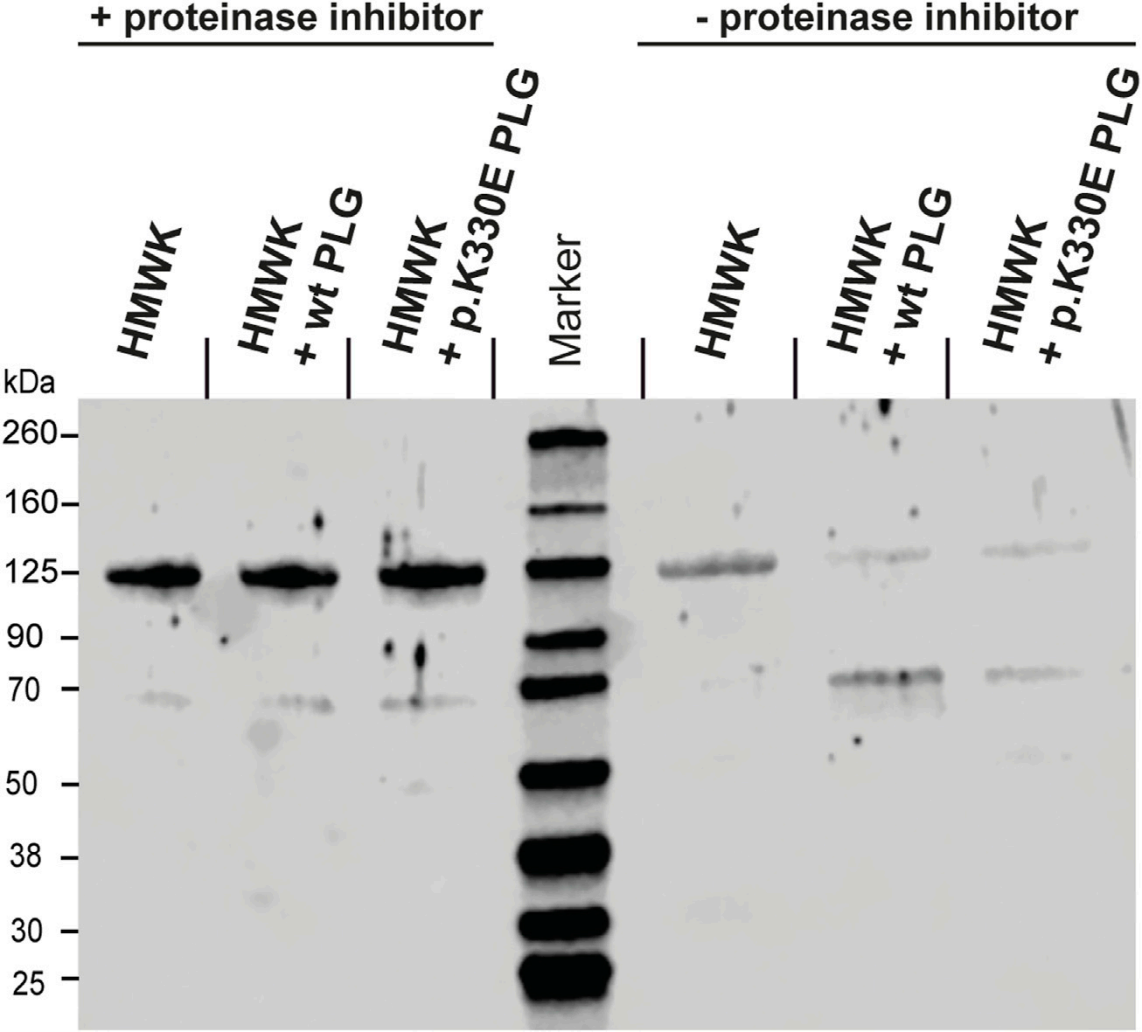
Western blot after 22 h of incubations showing HMWK incubated alone, with wt plasminogen, and with mutant plasminogen (p.K330E) in the presence of proteinase inhibitor (left side of the blot) and without proteinase inhibitor (right side).

Next, we wanted to investigate if plasminogen is capable of cleaving HMWK *in vivo* and if the mutant is having a different activity in doing so. We incubated either purified HMWK alone, HMWK plus wt plasminogen, or HMWK plus mutant plasminogen at 37°C for different durations. To test if any degradation depends on proteinase activity, we did this incubation in parallel with and without proteinase inhibitor. Using SDS-Page and Western blot analysis we investigated the cleavage efficacy. We observed that all three samples showed no degradation when incubated in the presence of proteinase inhibitor (Figure 4, left side). Without proteinase inhibitor HMWK alone showed a weaker band at ∼125 kDa band, indicating self-cleavage activity. The signal of both HMWK samples incubated with plasminogen decreased drastically compared to HMWK alone, indicating a cleavage activity of both, wt and mutant plasminogen. There was an additional band at ∼70 kDa in these samples, which was stronger if incubated with wt plasminogen compared to mutant plasminogen (Figure 4, right side), indicating an increased HMWK cleavage activity in mutant plasminogen compared to wt plasminogen.

**FIGURE 5:**
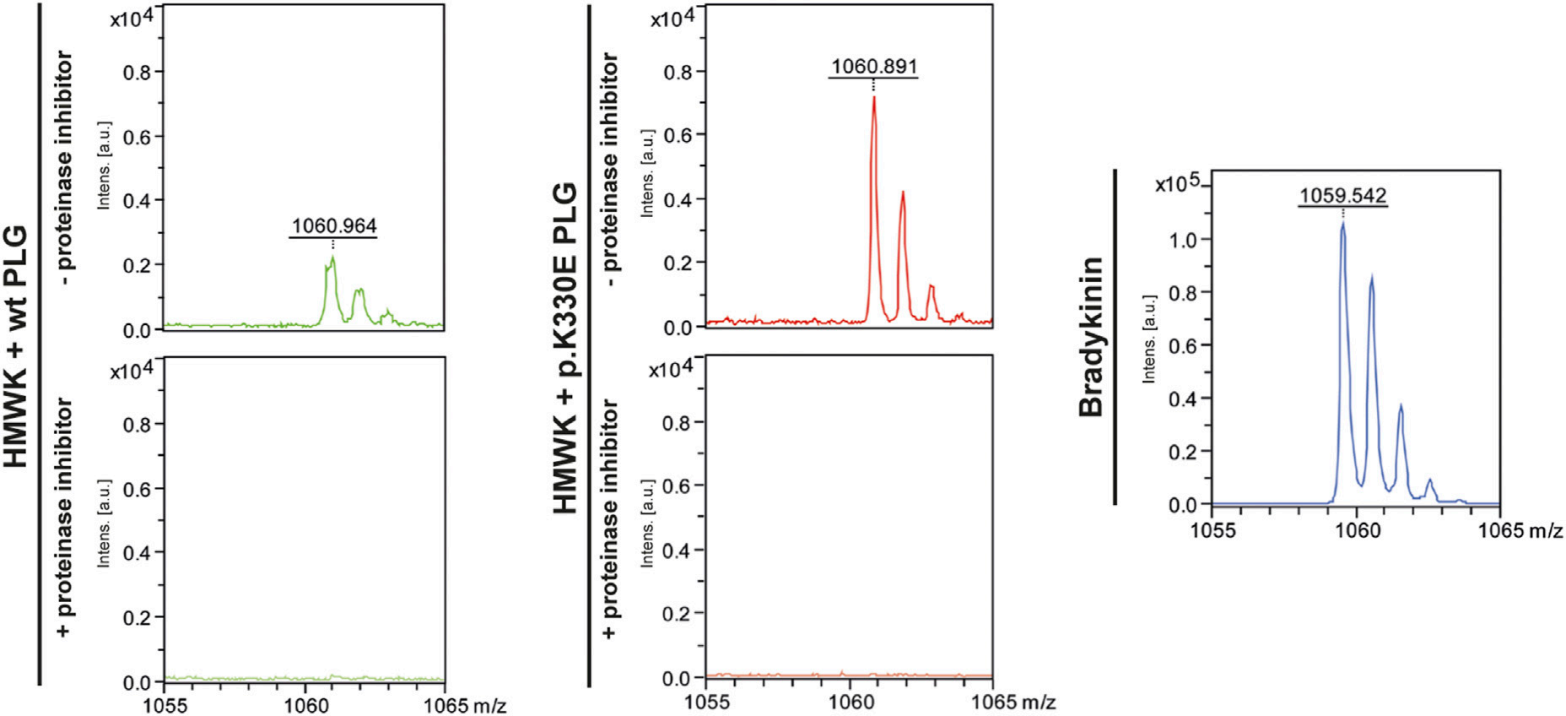
MALDI-TOF measurements for HMWK incubated with wt plasminogen and with mutant plasminogen (p.K330E) in the presence of proteinase inhibitor and without proteinase inhibitor, including the measurement of purified bradykinin as positive control (lower panel).

To investigate if this increased cleavage of HMWK also results in the generation of bradykinin, we analyzed these samples with MALDI-TOF-MS (Matrix-Assisted Laser-Desorption/Ionization Time-Of-Flight Mass-Spectrometry) to monitor the formation of the bradykinin molecule. Samples of HMWK incubated without proteinase inhibitor revealed distinct mass signatures at the calculated weight of bradykinin (1060.56). Control measurements with synthesized bradykinin showed a similar pattern. The presence of proteinase inhibitor resulted in the total absence of any bradykinin signal (Figure 5).

## Discussion

Since the identification of *PLG* mutations in HAE (HAE-PLG) (Bork et al., 2018), the pathomechanism how mutant plasminogen results in HAE has not been solved completely. There are indications for bradykinin involvement (Bork et al., 2020), and a recent study did not only demonstrate the possibility of plasminogen to directly cleave kininogens to release bradykinin, but also the increased efficiency of mutant plasminogen to do so (Dickeson et al., 2022). Yet, it was still not solved completely whether the KKS cascade is involved in bradykinin overproduction in HAE-PLG.

Here, we identified in a big HAE-PLG family individuals carrying an additional *F12* mutation, effectively resulting in the loss of FXII expression from one allele, and thus is reducing the presence of FXII protein at least by half. FXII plays an important role in the KKS: activated FXII (FXIIa) converts PK to kallikrein, which cleaves HMWK to release bradykinin. We hypothesized that, if mutant plasminogen acts *via* the KKS, HAE patients with reduced amounts of FXII would have a milder phenotype, as the loss of FXII would counteract the effects of the plasminogen mutation. Clinical comparison of patients carrying only the *PLG* mutation with patients carrying the additional *F12* mutation on top of the *PLG* mutation revealed that the presence of the additional *F12* did have no impact on the clinical presentation of the HAE-PLG patients. This gave a clear indication that the pathology in HAE-PLG might not be achieved *via* the KKS.

The next question was how the *PLG* mutation could result in increased bradykinin production, if it is not *via* the KKS. About 3 decades ago it has been shown that plasminogen is in principle capable of cleaving HMWK (Kleniewski and Donaldson, 1987; Kleniewski et al., 1992). Interestingly, 8 years before the *PLG* mutation p.K330E was identified to be causative for HAE (Bork et al., 2018), the plasminogen amino acid K330 had been investigated (Christen et al., 2010). This position within the Kringle domain 3 of plasminogen is broadly conserved amongst vertebrates, however as glutamic acid (E, which is the mutation found in HAE-PLG). The authors concluded that at some point during human evolution glutamic acid (E) was mutated to lysine (K). They could show that reverting it to glutamic acid increased docking of lysine-like zwitterionic ligands (Christen et al., 2010). This suggests that plasminogen p.K330E could be a gain-of-function mutation. This was confirmed by a recent *in vitro* study showing that mutant plasminogen releases bradykinin more effectively than wild type plasminogen (Dickeson et al., 2022). Modeling of p.K330E mutation indicates a widening within the ligand binding groove of the Kringle domain 3, which is in accordance with earlier modeling attempts of this domain (Christen et al., 2010). This likely allows for an altered proteolytic activity and is in line with the gain-of-function theory. We could confirm the gain of function by the p.K330E mutation *in vitro* using purified HMWK which we incubated with purified wild type respectively mutant plasminogen. Mutant plasminogen did not only cleave HMWK more efficiently, but also resulted in an increased bradykinin production compared to wild type plasminogen. We observed more intense signals at the bradykinin mass in the mutant plasminogen group in all experiments.

In summary, the clinical, structural, and biochemical data lead us to the conclusion that the HAE-causing plasminogen mutation p.K330E results in a gain of function, which enables mutant plasminogen to cleave HMWK more efficiently than the wt protein and produce a critical amount of bradykinin by bypassing FXII/kallikrein. Thus, treatment approaches for HAE-PLG should be aimed directly at plasminogen.

## Data Availability

The original contributions presented in the study are included in the article/supplementary material, further inquiries can be directed to the corresponding author.
